# Pregnancy with giant ovarian dysgerminoma: A case report and literature review

**DOI:** 10.1097/MD.0000000000021214

**Published:** 2020-10-09

**Authors:** Xi-Wen Zhang, Li-Rong Zhai, Dong-Wei Huang, Zhen-De Jiang, Tong Yu, Shu-Yan Liu, Man-Hua Cui

**Affiliations:** a Department of Gynecology; b Department of Gynecology and Obstetrics, Peking University People Hospital, Beijing; c Department of Pathology; d Department of Orthopaedics; e Department of Ophthalmology, The Second Hospital of Jilin University, Changchun, Jilin Province, China.

**Keywords:** chemotherapy, dysgerminoma, malignant germ cell tumor, pregnancy, pregnancy outcome, surgery, treatment

## Abstract

**Rationale:**

Dysgerminoma is an extraordinarily rare neoplasm arising from the malignant germ cells of the ovary. Early antenatal diagnosis and proper management of the neoplasm to improve maternal-neonatal results are the considerable challenges facing the gyne-oncologist. We summarize the clinical features and discuss treatment strategies of the ovary dysgerminoma (OD). Besides, we also review the literature on OD in PubMed, Web of Science Core Collection, Library of Congress, and LISTA from 1939 to 2019 to evaluate its clinical characteristics, feto-maternal compromise, management, and fertility outcome.

**Patient concerns:**

A 25-year-old pregnant woman reported lower abdominal pain and vomiting.

**Diagnosis:**

The patient was diagnosed as right OD.

**Interventions:**

She received a cesarean section due to severe abdominal pain, delivered a healthy girl at 38 C 4 weeks of gestation, and accepted fertility-preserving surgery. However, the patient refused chemotherapy postoperatively.

**Outcomes:**

The patient was followed up 42 days, 3 months, and 6 months after surgery, and no tumor recurrence was observed.

**Lessons:**

OD has non-specificity characteristics, including age, symptoms, image date, and tumor marks. However, these abnormal indicators may provide some evidence for accurate antenatal diagnosis. The management strategies should be considered comprehensively on an individual basis, and fertility-preserving surgery should be carried out in the second trimester if further pregnancy is desired. Adjuvant chemotherapy needs to be applied to the treatment of OD patients with The International Federation of Gynecology and Obstetrics (FIGO) stages II, III, and IV and timely chemotherapy is suggested if there are several weeks before the expected date of delivery. The overall prognosis of OD patients is excellent.

## 1 Introduction

Malignant germ cell tumor (MGCT) is an extraordinary rare ovarian cancer, which occupies no >5% of all ovarian cancers^[1–4]^ and 18% to 26% of all ovarian cancer with pregnancy.^[5,6]^ MGCT mainly includes the following subtypes: ovary dysgerminoma (OD) (38.2%), yolk sac tumor (30.4%), and immature teratoma (15.7%).^[2]^ OD is the most common subtype of MGCT and often occurs in adolescence and early adulthood.^[1,7–10]^ In pregnant women, OD patients only account for about 0.0002% to 0.001%,^[11]^ and OD usually has a unilateral onset and is diagnosed at an early stage. It is difficult to achieve a large sample of OD due to its relatively low incidence. Thus, more studies are needed to summarize the clinical features and determine the optimal management strategies of OD. Furthermore, OD associated with mental retardation in pregnant women is even rarer. Therefore, the purpose of this study is to report our seldom case, as well as to review the literature on OD features, differential diagnosis, management strategies, and prognosis of pregnant patient with OD.

## 2 Ethic

This case report was approved by the institutional review board of the second hospital of Jilin University. Informed written consent was obtained from the patient for publication of this case report and accompanying images.

## 3 Methods

We report a case of OD with mental retardation and review relevant literature in PubMed, Web of Science Core Collection, Library of Congress, and LISTA from 1939 to 2019 (Table 1).

**Table 1:**
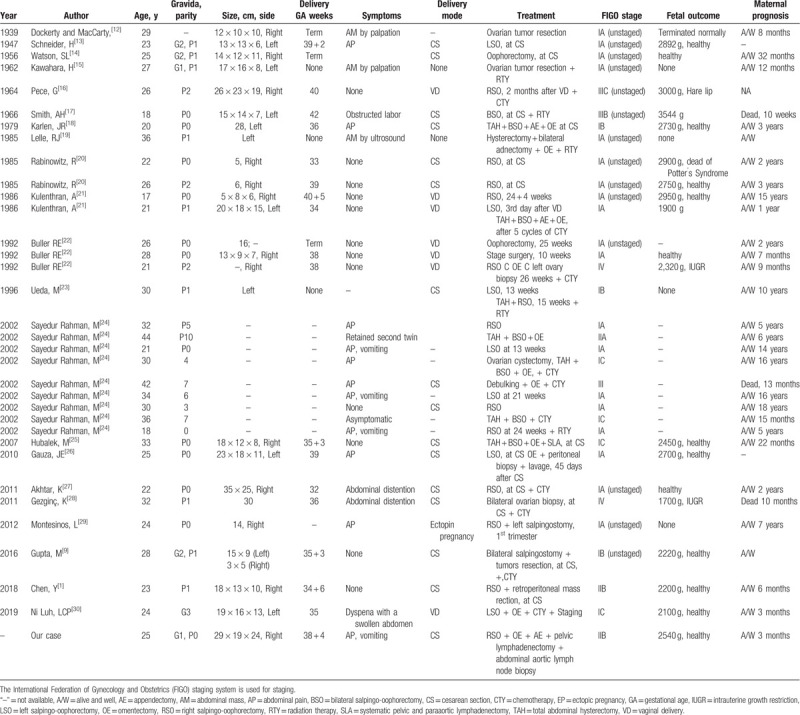
Clinical features in 34 cases of ovarian dysgerminoma in pregnancy.

## 4 Case report

**Figure 1:**
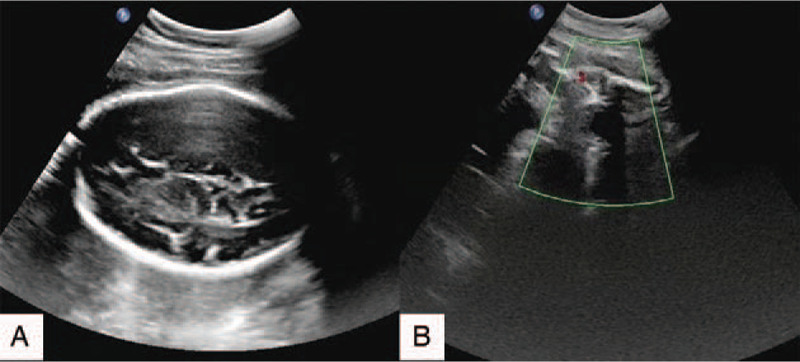
Ultrasound showed a hypoechoic mass behind the uterus, about 23.0 × 12.5 cm in size.

A 25-year-old pregnant woman with mental retardation who had abdominal pain and vomiting for 7 hours was transferred to our department. The previous history was gravida 1, para 0, without surgery history. Her initial prenatal examination was performed at 12 weeks of gestation. The ultrasound indicated pregnancy status and revealed a large mass in the pelvic cavity. The regular ultrasound examination during pregnancy revealed that the volume of the mass increased gradually. At 30 C 2 weeks of gestation, the ultrasound revealed cephalic presentation. The biparietal diameter (BPD) was 6.9 cm, the head circumference (HC) was 26.6 cm, the abdominal circumference (AC) was 24.7 cm, and the femur length (FL) was 5.6 cm. The posterior wall of the placenta was grade I and the lower margin was 1.6 cm from the inner cervix. The amniotic fluid index (AFI) was 16.1. The ultrasound also revealed a hypoechoic mass in the lower part of the posterior wall of the uterus with a size of 14.8 cm × 8.5 cm. At 38 C 4 weeks of gestation, the ultrasound before admission of the patient revealed cephalic presentation. The BPD was 7.8 cm, HC was 31.5 cm, AC was 32.9 cm, and FL was 6.8 cm. The right wall of the placenta was late grade II and AFI was 12.2 cm. A U-shaped impression was found on the neck of the fetus. The ultrasound also revealed a hypoechoic mass located at the right rear of the uterus with a size of 23.0 cm × 12.5 cm (Fig. 1). Some of her tumor markers were positive. Human chorionic gonadotropin (HCG) was 14,333.94 mIU/mL (0–5 mIU/mL), the α-fetoprotein (AFP) was 142.59 ng/mL (0–8.78 ng/mL), Cancer antigen (CA)-125 was 148.10 U/mL (0–35 U/mL), CA-199 was 610.46 U/mL (0–37 U/mL), CA-50 was 59.10 U/mL (0–20 U/mL), Cytokeratin 19 fragment was 4.86 ng/mL (0–2.08 ng/mL), and neuron-specific enolase (NSE) was 76.04 ng/mL (0–15 ng/mL). Conversely, some of her tumor markers were negative, such as carcinoembryonic antigen (CEA), CA-153, and squamous cell carcinoma antigen (SCC).

On abdominal examination, the uterine fundal height was 33 cm and the abdominal circumference was 98 cm. The abdominal tenderness was positive, especially in the right lower abdomen, and the rebound tenderness was also positive. The patient could not cooperate in the other examination.

Termination of pregnancy was performed due to severe abdominal pain. She delivered a 2540 g healthy girl with a 1-minute Apgar score of 9 by cesarean section (CS) and a 10-minute Apgar score of 10 by CS.

**Figure 2:**
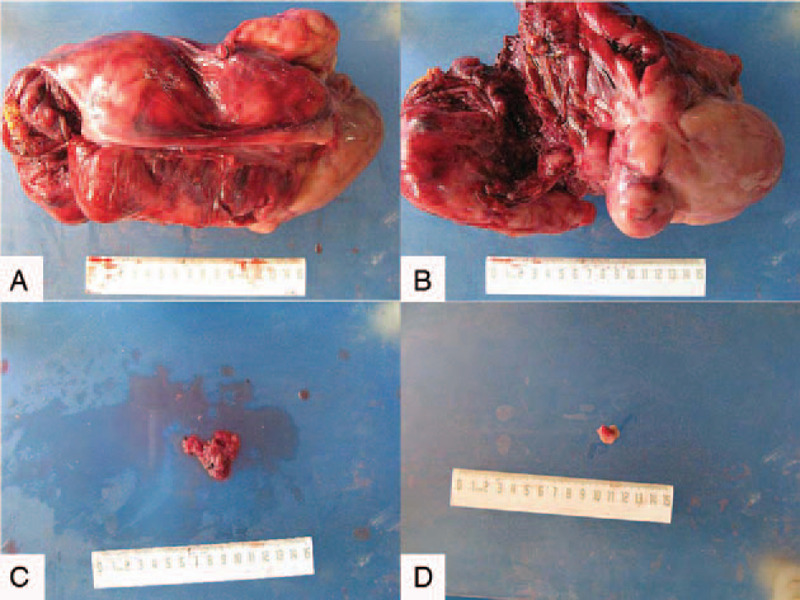
(A and B) Gross appearance of tumors resected. (C) Tumor biopsy. (D) Contralateral ovarian biopsy.

Intraoperatively, we found a large solid mass of 25 cm × 19 cm × 24 cm, which originated from the right ovary, with a moderate amount of pale-yellow ascites. The tumor was substantially lobulated, the texture was soft, the surface was intact, and the tissue was crunchy. Large blood vessels were visible, and the boundary between the tumor and adjacent organs (the right lining of the uterus, the rectal serosa) was not clear. No abnormalities in the appearance of the ovaries and fallopian tubes were found. At sectioning (Fig. 2), the mass was grayish-white, grayish-yellow, grayish red, and homogeneous. The tumor was almost solid, while some areas were soft, of which the density was similar to brain medulla. No enlarged lymph nodes were found in the pelvis and abdominal cavity. The right fallopian tube was 7 cm long and 0.3 to 0.7 cm in diameter. Tumor biopsy and contralateral ovarian biopsy were conducted primarily.

The pathological results of the intraoperative frozen section showed right adnexa dysgerminoma; left ovarian biopsy showed no tumor, but localized old bleeding and interstitial fibrosis. Fertility-preserving surgery, including giant tumor and right adnexa resection, omentectomy, appendectomy, pelvic lymphadenectomy, abdominal aortic lymph node biopsy, was performed.

**Figure 3:**
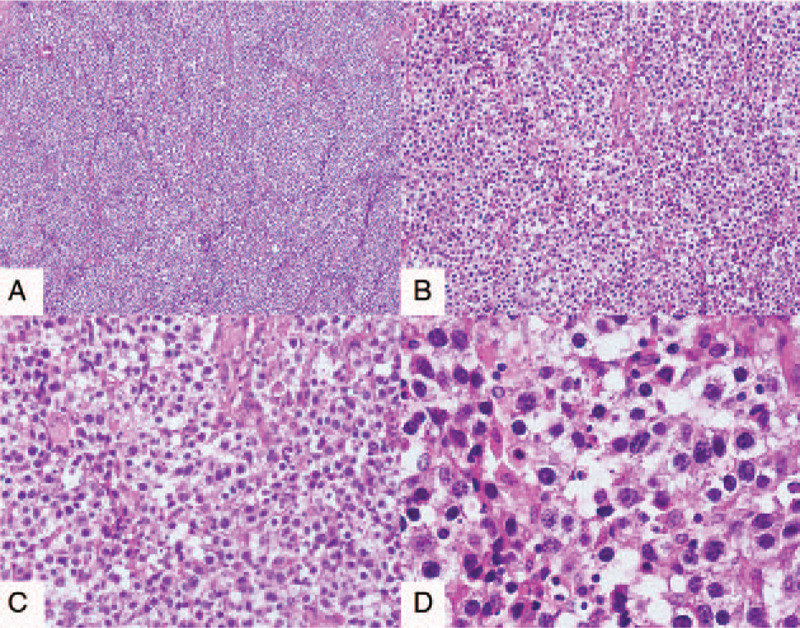
Hematoxylin-eosin (HE) staining results of the adnexa dysgerminoma, consist of round to atypical oval cell separated with complex thin fibrous tissue septal network which has rich lymphocyte infiltrate. (A ×40, B ×100, C ×200, D ×400).

**Figure 4:**
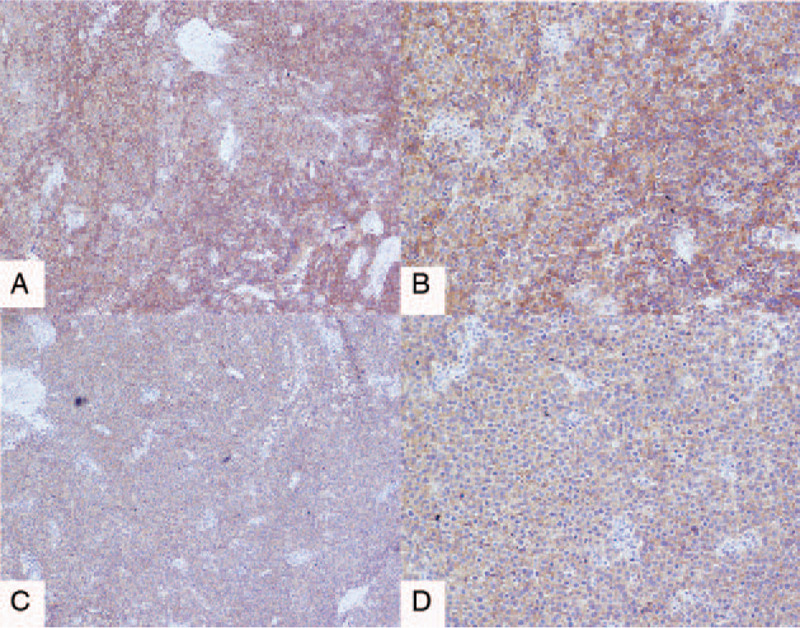
Immunohistochemical staining results of the adnexa dysgerminoma. (A and B) D2–40 staining was positive (A, D2–40, ×40, B, D2–40, ×100). (C and D) CD177 staining was positive (C, CD177, ×40, D, CD177, ×100).

**Figure 5:**
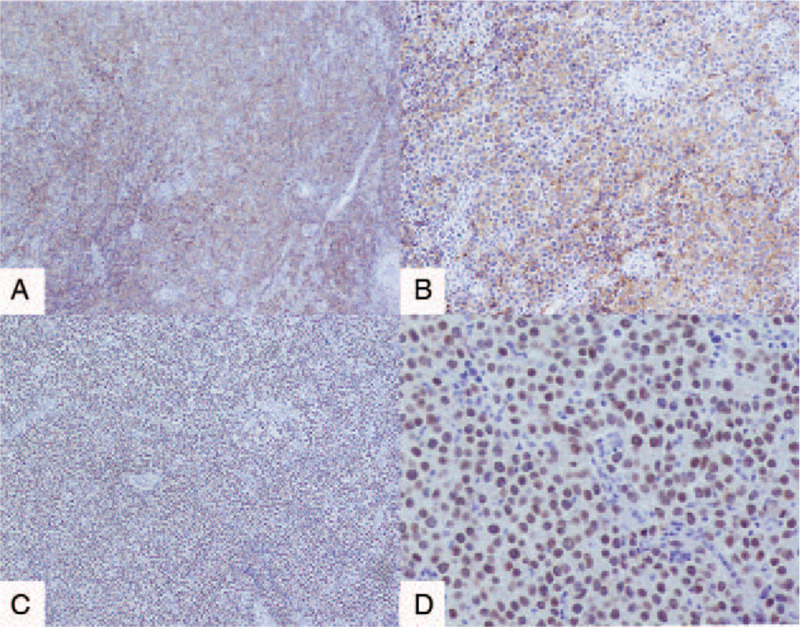
Immunohistochemical staining results of the adnexa dysgerminoma. (A and B) PLAP staining was positive (A, PLAP, ×40, B, PLAP, ×100). (C and D) SALL4 staining was positive (C, SALL4, ×40, D, SALL4, ×100).

**Figure 6:**
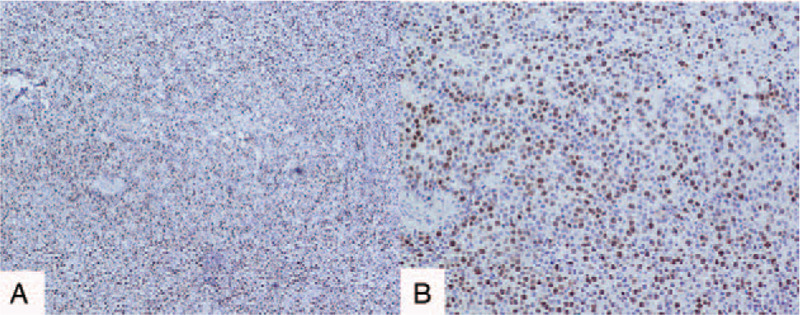
Immunohistochemical staining results showed that the positive index of Ki67 was 70%.

The final pathological results showed dysgerminoma (Fig. 3A–D) of the right adnexa, but no tumor metastasis was found in the right fallopian tube, left ovary, appendix, omentum, and pelvic lymph nodes. Immunohistochemical (IHC) results were as follows: D2–40, CD117, PLAP, and SALL-4 were positive (Figs. 4 and 5); CK (AE1/AE3), Vimentin, epithelial membrane antigen (EMA), estrogen receptor (ER), progesterone receptor (PR), Alpha fetoprotein (AFP), and Glypican-3 were negative, and the positive index of Ki67 was 70% (Fig. 6). The final clinical diagnosis of the patient was OD, stage IIB (according to the 2014 FIGO staging system).

The patient's postoperative vital signs were stable, and the incision healed well. However, the patient refused chemotherapy postoperatively. The follow-up results of the patient 42 days, 3 months, and 6 months after the surgery showed no tumor recurrence.

## 5 Discussion

OD is the most common subtype of MGCT, which originates from ovarian primordial germ cells. It often occurs in adolescence and early adulthood, but has been found only about 0.0002% to 0.001% of pregnant women.^[11]^ It is hard to achieve a large sample of OD due to its extraordinary low incidence. Therefore, this paper is aimed to report our rare case, as well as to review the relevant literature summarizing the features, differential diagnosis, management strategies, and prognosis of pregnant patients with OD.

### 5.1 Features of ovarian dysgerminoma

OD can occur in women aged from 7 months to 70 years,^[31]^ but predominantly in young pregnant women.^[1,7,8,30,32]^ The majority of OD pregnant women usually have non-specific symptoms,^[33]^ including the most common abdominal pain (35.3%), followed by abdominal distention (19.6%), a growing mass (19.6%), multiple symptoms (18.6%), and non-symptoms (21.6%).^[2]^ In our study, abdominal pain was the main complaint of the patient and led to a cesarean section.

Considering the gross pathologic features of OD, it usually presents well encapsulated and characteristically solid, with a diameter range from 8 to 15 cm.^[25,31]^ At sectioning, the tissue is lobulated, soft, fleshy, and gray-white or light tan. Occasionally, areas of hemorrhage and coagulative necrosis, which are typically related to cystic changes, can be observed. OD is most commonly unilateral in pregnancy, accounting for approximately 95%,^[1]^ while only 5% to 20% is bilateral.^[1,34–36]^ In our study, the tumor was unilateral and showed substantially lobulated soft texture and the entire surface. This finding was consistent with the previous literature.^[1,25,31–33]^

Regarding the microscopic pathologic feature of OD, it is like that of testicular seminomas. OD is composed of round cells with a uniform population, which is usually infiltrated by T lymphocytes and separated by fibrous strands. A large round or flattened nucleus that contains one or a few prominent nucleoli and clear eosinophilic cytoplasm can be observed in the center of cells. In addition, mitoses are always in large quantities.^[31]^

Regarding the imaging features of OD, it is characterized by pure solids. In ultrasonography, they show well-defined borders, smooth lobulated contours, and component lobules, with heterogeneous echogenicity. At Doppler ultrasonography, they are abundantly vascularized at power and color.^[37–39]^ In our study, ultrasound results show unclear boundaries, component lobules, with heterogeneous echogenicity. This feature suggests that the mass may be malignant. At CT, the lobular pattern may also be observed with a predominantly solid tumor accompanied by enhancing septa and areas of cystic change.^[38,40]^ Kim and Kang^[38]^ claimed that calcification might be shown as a speckle. In magnetic resonance imaging (MRI), the most characteristic appearance is a solid mass, which is divided into lobules by fibrovascular septa. On T2-weighted images, the signal intensity is isointense or slightly hyperintense. On T1-weighted images, the signal intensity of OD is lower than that of muscles. Kitajima et al^[41]^ described that the MRI features of epithelial ovarian neoplasms were similar to those of multilocular cystic masses with irregular septations. Unfortunately, this patient did not undergo CT and MRI examinations during the hospitalization.

Considering the tumor marks of OD, CA125, and NSE may provide reliable evidence in OD.^[42]^ Literature reported that high levels of serum CA125 rapidly fell after chemotherapy.^[25]^ Previous studies described that partly OD patients exhibited increased NSE content and positive NSE of IHC^[43,44]^ The serum levels and IHC expression of NSE in pediatric patients with OD may be of value in patient monitoring.^[42]^ In this study, CA125 and NSE increased significantly preoperatively. Some other indicators are abnormal, including HCG, AFP, CA-199, and CA-50. We hope that these positive indicators can provide some help for other scholars to diagnose OD accurately. Besides, LDH is another reliable indicator for predicting the effect of chemotherapeutic intervention.^[25,45]^

### 5.2 Differential diagnosis of ovarian dysgerminoma

OD has nonspecific features, which lead to the difficulty in making an accurate diagnosis. However, the age of patients, the imaging features of the neoplasm, and the abnormal tumor markers may help to determine a correct differential diagnosis. In general, OD should be distinguished from other purely solid masses of ovarian, including fibrosarcomas, granulosa cell tumors, Brenner tumors, epithelial ovarian, and metastatic carcinomas.^[46]^

### 5.3 Treatment strategies of ovarian dysgerminoma

With regard to surgery treatment of OD, accurate surgical staging is relatively critical to determination of the reasonable and accurate risk-based management. Currently, the FIGO classification is the most accepted method.^[47]^ OD staged IA-C could achieve acceptable surveillance by fertility-sparing unilateral salpingo-oophorectom.^[31]^ Bilateral salpingo-oophorectomy and hysterectomy are recommended for stage II and III diseases. In addition, if tumors do not invade the contralateral reproduction organs, unilateral salpingo-oophorectomy can be considered. The management strategies of stage IV patients mainly include fertility-sparing surgery, cytoreduction, and adjuvant chemotherapy.^[48,49]^ Regarding second-look surgery of OD, if the tumor contains teratomatous elements or has residual disease, patients may benefit from second-look surgery after initial cytoreductive surgery and chemotherapy.^[48,50]^ However, if the tumor does not have a teratomatous element, <5 cm of residual disease, or normal tumor marker levels after chemotherapy, second-look surgery is not recommended.^[50]^

In this study, the patient's mental retardation and lack of awareness of contraception may lead to repregnancy and increase the family burden. Thus, the patient's guardian strongly requests hysterectomy and bilateral appendectomy for the patient. However, the patient does not meet the indications for hysterectomy and bilateral appendectomy according to the FIGO stage, age, and grade of malignancy. Also, in China, especially in rural areas, women who have lost fertility function are not competitive in the remarriage population. What is worse, she has mental retardation, which means that it is difficult for the patient to reconstitute a family once she divorces after hysterectomy and appendectomy. Therefore, after careful consideration, we performed fertility-preserving surgery for the patient. The patient showed a satisfactory treatment effect during the follow-up visit.

With regard to chemotherapy of OD, OD with FIGO stages II, III, and IV are indicated for chemotherapy.^[47]^ Chemotherapy is recommended based on pathological evidence,^[1]^ especially in cases with advanced-stage tumors, mixed epithelial and germ cell tumors, large tumor size, and rapidly increasing ascites. To date, platinum-based chemotherapy is the main strategy, including paclitaxel-carboplatin (TC) and bleomycin-etoposide-cisplatin (BEP).^[25,51–56]^ In 2004, Hubalek et al^[25]^ claimed that TC could elicit an excellent response and posed no adverse impacts on the fetus. BEP is usually applied to the treatment of nonepithelial ovarian tumors of nonpregnant patients. However, the incidence of adverse advents (plagiocephaly, fetal ventriculomegaly with cerebral atrophy, hearing loss, and syndactyly) of etoposide is high.^[57–60]^ Therefore, in pregnancy, paclitaxel-carboplatin chemotherapy instead of BEP is an optimized scheme for the treatment of nonepithelial ovarian cancer.^[61]^ The influence of chemotherapy during pregnancy on maternal survival must be considered. Literature^[62,63]^ reported that chemotherapy during the first trimester could increase the incidence of fetal death, abortion, and malformations. Furthermore, the study also showed that the central nervous system, hemopoietic system, the eyes, and genitals were still vulnerable to sustained exposure to antineoplastic agents after organogenesis.^[64]^ However, increasing evidence suggests that chemotherapy for the second and third trimesters is relatively safe.^[65]^

In our study, the patient was diagnosed as OD staged II B, and chemotherapy was recommended by gynecologists postoperatively. However, she refused. Optimistically, there was no recurrence during the follow-up period of 6 months. We attribute this positive outcome partly to the low malignancy of the tumor and the standard and thorough operation carried out by a gynecologist with >30 years of experience, and partly to the short follow-up period.

### 5.4 Prognosis of ovarian dysgerminoma

Residual disease, tumor markers, the FIGO stage, and the volume of the residual tumor are all the critical factors of prognosis.^[48]^ Besides, age over 45 years is also a significant predictor of recurrence.^[49]^ In most cases, tumors are detected early, which contribute to accurate prognosis.^[66]^ The prognosis of early-stage OD patients is excellent,^[48,49,67]^ and the overall 5-year survival rate is approximately 100%.

## 6 Conclusion

In conclusion, features of OD, including age, symptoms, images date, and tumor marks, have non-specificity. However, these abnormal indicators may provide some evidence for accurate antenatal diagnosis. The management strategies should be considered comprehensively on an individual basis, and fertility-preserving surgery should be carried out in the second trimester if further pregnancy is desired. Adjuvant chemotherapy needs to be applied to the treatment of OD with FIGO stages II, III, and IV. If there are several weeks before the expected date of delivery, timely chemotherapy is indicated. The overall prognosis of OD patients is excellent.

## Author contributions

**Conceptualization:** Tong Yu, Shu-Yan Liu, Man-Hua Cui.

**Data curation:** Xi-Wen Zhang, Li-Rong Zhai, Dong-Wei Huang, Zhen-De Jiang.

**Formal analysis:** Xi-Wen Zhang, Dong-Wei Huang, Zhen-De Jiang.

**Methodology:** Li-Rong Zhai, Zhen-De Jiang, Shu-Yan Liu.

**Resources:** Dong-Wei Huang.

**Supervision:** Tong Yu, Man-Hua Cui.

**Writing – original draft:** Xi-Wen Zhang, Li-Rong Zhai.

**Writing – review & editing:** Tong Yu, Shu-Yan Liu, Man-Hua Cui.

## Abbreviations

Abbreviations: AC = abdominal circumference, AFI = amniotic fluid index, AFP = α-fetoprotein, BEP = bleomycin-etoposide-cisplatin, BPD = biparietal diameter, CA = cancer antigen, CEA = carcinoembryonic antigen, CS = cesarean section, FIGO = The International Federation of Gynecology and Obstetrics, FL = femur length, HC = head circumference, HCG = human chorionic gonadotropin, IHC = immunohistochemical, LDH = lactic dehydrogenase, MGCT = malignant germ cell tumor, NSE = neuron-specific enolase, OD = ovarian dysgerminoma, SCC = squamous cell carcinoma antigen, TC = paclitaxel-carboplatin.
